# Integrative taxonomy elucidates phylogenetic position of a clawless African eutardigrade (Tardigrada) supporting the erection of a new genus

**DOI:** 10.1038/s41598-025-17679-7

**Published:** 2025-10-03

**Authors:** Jędrzej Warguła, Wiktoria Dmuchowska, Daniel Stec, Łukasz Kaczmarek

**Affiliations:** 1 Department of Animal Taxonomy and Ecology, Adam Mickiewicz University in Poznań, Uniwersytetu Poznańskiego 6, Poznań, 61-680 Poland; 2 Department of Animal Morphology, Adam Mickiewicz University in Poznań, Uniwersytetu, Poznańskiego 6, Poznań, 61-680 Poland; 3 Institute of Systematics and Evolution of Animals, Polish Academy of Sciences, Sławkowska 17, Kraków, 31-016 Poland

**Keywords:** *Apodibius*, Barcodes, Hexapodibiidae, Soil, Tropical regions, Uganda, Evolutionary genetics, Phylogenetics, Taxonomy, Evolution, Genetics, Zoology

## Abstract

In the presented study, we re-investigated the soil-dwelling clawless tardigrade species *Apodibius nuntius* (Binda, 1984) using an integrative taxonomic approach. Our analysis is based on detailed morphological comparisons between three populations from three different countries: Mozambique (type population), Australia and Uganda. The first two populations comprise historical specimens deposited in museum collections, whereas the third one is newly found and reported for the first time in this study. In addition to morphological analysis, we constructed a phylogenetic tree of Isohypsibioidea using two ribosomal gene fragments (18S rRNA and 28S rRNA). The obtained results enabled us to establish a new genus, *Sinunguibius* gen. nov., which is assigned to the family Hexapodibiidae. The new genus constitutes the second known genus within Eutardigrada characterized by a complete absence of claws. The main morphological chacter that differentiates the new genus from the also clawless genus *Apodibius* is the number of placoids in the pharynx. Our results suggest that the complete loss of claws is a convergent trait that occurs in at least two families: Doryphoribiidae and Hexapodibiidae. The inclusion of the new genus within the family Hexapodibiidae required a corresponding amendment to its family diagnosis.

**Supplementary Information:**

The online version contains supplementary material available at 10.1038/s41598-025-17679-7.

## Introduction

Water bears (Tardigrada^1^, also known as moss piglets, are microinvertebrates recognized for their extraordinary resilience to extreme environmental conditions, especially in the state of cryptobiosis. This capability allows tardigrades to withstand environmental extremes such as severe desiccation, prolonged freezing, intense radiation, fatal chemicals, high and low pressure or even vacuum conditions^2–9^.

Despite the high biodiversity of the ecosystems in Uganda^10 ^many groups of invertebrates are still very poorly known. This is especially true for challenging group of microinvertebrates such as the phylum Tardigrada, which diversity and distribution in entire Africa is understudied^11–17^. In Uganda, tardigrades were reported mostly from mountain ecosystems and so far, only 14 genera all comprising 17 species, in total, are known in this region^15,18,19^.

Limno-terrestrial tardigrades are those that inhabit various substrates such as mosses, lichens, leaf litter, sediments or soil^20^. Interestingly, representatives of soil-dwelling tardigrades are often characterized by a significant reduction of claws (genera *Xerobiotus* Bertolani & Biserov, 1996^21^, *Hexapodibius* Pilato, 1969^22^) or even their complete absence on some or on all legs (genera *Necopinatum* Pilato, 1971^23^, *Apodibius* Dastych, 1983^24^). Claws reduction is most likely related to the soil environment, where it facilitates movement between small grains of soil^21,25–28^. The process of claw reduction can be considered convergent, as it is observed in several families of eutardigrades within the order Parachela^26,28–30^ such as: Calohypsibiidae Pilato, 1969^22^, Doryphoribiidae Gąsiorek, Stec, Morek & Michalczyk, 2019^31^, Eohypsibiidae Bertolani & Kristensen, 1987^32^, Hexapodibiidae Cesari, Vecchi, Palmer, Bertolani, Pilato, Rebecchi & Guidetti, 2016^29^, Isohypsibiidae Sands, McInnes, Marley, Goodall-Copestake, Convey & Linse, 2008^33^ and Macrobiotidae Thulin, 1928^34^.

The genus *Apodibius* was erected in 1983, with the inclusion of one species *Apodibius confusus* Dastych, 1983^24^ discovered in Poland^24^. The genus and also the species’ most distinctive character, was the absence of claws on all legs. Since then, only two other species have been described within this genus i.e., *Apodibius richardi* Vargha, 1995^35^ from Hungary and *Apodibius nuntius* Binda, 1984^36^ from Mozambique. Importantly, there was a fourth species, *Apodibius serventyi* Morgan & Nicholls, 1986^37^ described from Australia, but it was later synonymized with *Apo. nuntius* by Van Rompu et al., 1994^38^. The morphology within this genus in not uniform regarding the placoid number in the pharynx, since two species, *Apo. confusus* and *Apo. richardi*, are characterized by the presence of two macroplacoids in the pharynx, while *Apo. nuntius* has three. Therefore, considering the significance of the number of macroplacoids in eutardigrade taxonomy^39,40 ^it can be assumed that the genus *Apodibius* is polyphyletic.

Claws are considered one of the most important characters for tardigrade classification^22,41^. Thus, the clawless genus *Apodibius* was classified as *incertae sedis*^24^ until 2014, when the first DNA sequences of *Apo. confusus* were published, and subsequent phylogenetic analysis resulted in the assignment of the genus to the family Isohypsibiidae^42^. Ultimately, thanks to the broader taxonomic and phylogenetic sampling, the genus *Apodibius* was transferred to newly erected family Doryphoribiidae by Gąsiorek et al., 2019^31^.

In this paper, we analysed genetically and morphologically newly collected specimens of *Apo. nuntius* from a sample of moss with a large amount of soil collected on the Kalyango Hills, Kabarole district in Western Region of Uganda. Additionally, we also examined historical specimens of this species that are stored in museum collections and originate from Mozambique (type population) and Australia. Based on analyses of two ribosomal markers (18 S rRNA and 28 S rRNA) and the species’ morphological distinctiveness, we established a new genus within the family Hexapodibiidae to accommodate the studied taxon.

## Materials and methods

### Samples and sample processing

A moss sample with a large amount of soil was collected at Kalyango Hills in Western Region (Kabarole District) of Uganda in July 2023. The sample was packed in a paper envelope, dried at the temperature of ca. 25 °C and transported to the laboratory at the Faculty of Biology, Adam Mickiewicz University in Poznań (Poland). Tardigrades were extracted from the sample and studied following the protocol by Stec et al., 2015^43^. Three eutardigrades were extracted from the sample, out of which two were directly mounted in permanent microscope slides while the third one was destined for genetic analysis.

### Microscopy and imaging

Specimens for morphological analyses were mounted on permanent microscope slides in Hoyer’s medium, secured with cover slips. Slides were dried in a heater for two days at 60 °C and sealed with transparent nail polish. Later, slides were analysed using an Olympus BX41 Phase Contrast Light Microscope (PCM) equipped with an Olympus SC50 digital camera (Olympus Corporation, Shinjuku-ku, Japan). Photomicrographs were compiled using GIMP 2.10.36. For deep structures that could not be adequately shown in a single image, a series of 2–10 photomicrographs were taken at approximately 0.5 μm intervals. These images were then manually merged into a single deep-focus image using GIMP 2.10.36.

### Morphometrics and morphological nomenclature

All measurements are given in micrometres [µm]. Structures were measured only if they were not damaged and their orientation was suitable. Body length was measured from the anterior extremity to the end of the body. Measurements of the buccal tube length and the position of the stylet support insertion point were performed according to Pilato, 1981^44^. The *pt* index was calculated as the ratio of the length of a specific structure to the length of the buccal tube, expressed as a percentage Pilato, 1981^44^. Remaining measurements of the buccal tube were performed according to Kaczmarek & Michalczyk, 2017^45^. The macroplacoid sequence following Kaczmarek et al., 2014^46^. The type of stylet furcae shape was classified following Pilato & Binda, 2010^47^. The nomenclature of peribuccal *papulae* following Camarda et al., 2024^27^. Genus abbreviation method following Perry et al., 2019^48^. Morphometric data were handled using modified “Parachela” ver. 1.8 template available from the Tardigrada Register^49^. Raw morphometric data for specimens, examined in this study are given in Supplementary Materials (SM.01–SM.03).

### Comparative material

In order to conduct a comparative morphological analysis of the newly found population, type specimens of *Apo. nuntius* from Mozambique (Slide No: 3637) was loaned from University of Catania (Via Androne, 81, 95124 Catania CT, Italy). Additionally, six specimens of *Apo. nuntius* from Australia (described originally as *Apodibius serventyi* Morgan & Nicholls, 1986; Slide No: WAM 86/34-9-53) were loaned from Western Australian Museum (49 Kew Street, Welshpool, WA 6106, Locked Bag 49, Welshpool DC, Australia).

### DNA extraction and genotyping

Before genomic DNA extraction, the specimen was identified in vivo using light microscopy. The DNA extraction was made using the Chelex^®^100 beads (Bio-Rad) extraction method^50 ^modified by Stec et al., 2020^51^. To obtain tardigrade exoskeleton, the remaining portion of the DNA extract containing Chelex^®^100 beads was diluted with ddH_2_O and transferred to a glass cube. The glass cube was examined under stereomicroscope and the exoskeleton was mounted in a permanent slide in Hoyer’s medium as described above.

We sequenced two molecular markers i.e., 18S rRNA, 28S rRNA which were amplified and sequenced using primers and protocols described in Stec et al., 2020^51^. The COI and ITS-2 amplification, using the same protocol and primers listed therein, were not successful for our sample. Sequencing products were read with the ABI 3130xl sequencer at the Genomed company (Warsaw, Poland). Sequence chromatograms were checked for accuracy using FinchTV 1.3.1 (Geospiza Inc.). Sequences were than processed in BioEdit ver. 7.2.5^52^ and submitted to GenBank.

### Phylogenetic analysis and genetic comparisons

To determine the phyletic position of the new genus, a phylogenetic tree was constructed based on the dataset used by Tumanov et al., 2024^53^, incorporating sequences obtained in this study, as well as, sequences published by Dabert et al., 2014^42^ and Gąsiorek et al., 2019^31^. However, we decided to limit the number of taxa by removing those with unpublished or uncertain status: *Eremobiotus* sp^54,55^. GenBank code: FJ435722, and *Doryphoribius* sp. GenBank code: MT872215 used previously in Tumanov et al., 2024^53^. The list of all sequences used in the analysis is provided in SM.04. The DNA sequences from the superfamily Macrobiotoidea were used as the outgroup. Sequence alignments were performed using the Q-INS-I method for both ribosomal markers (18 S rRNA and 28 S rRNA) in MAFFT version 7^56,57^. The alignments were then manually verified for non-conserved regions in BioEdit. After alignment, sequences were trimmed to lengths of 1008 bp (18S rRNA), 1317 bp (28S rRNA) and subsequently concatenated with SequenceMatrix^58^. Prior to partitioning, the concatenated alignment was divided into two blocks (one for each marker). PartitionFinder^59^ using the Akaike Information Criterion (AIC), was employed and as the optimal partitioning scheme retained two predefined partitions with the best model GTR + I + G for both markers. Maximum-likelihood (ML) reconstruction was conducted using W-IQ-TREE^60,61^ with a GTR + I + G model. One thousand ultrafast bootstrap (UFBoot) replicates were applied to provide support values for branches^62^. Bayesian inference (BI) was performed on the concatenated dataset (18 S rRNA + 28 S rRNA) using MrBayes v3.2^63^, with random starting trees. The analysis was run for 10 million generations, sampling every 1000 generations, and convergence was confirmed by an average standard deviation of split frequencies below 0.01. Tracer v1.6^64^ was used to ensure the Markov chains had reached stationarity and to determine the appropriate ‘burn-in’ period, which was the first 10% of generations. Effective Sample Size (ESS) values exceeded 200, and the consensus tree was obtained by summarizing the topologies after discarding the burn-in phase. The final tree was visualized using FigTree v1.4.3, available at http://tree.bio.ed.ac.uk/software/figtree (accessed on 10 August 2018). The raw trees are included in Supplementary Materials (SM.05).

## Results

### Taxonomic account of the new genus

Phylum: Tardigrada Doyère, 1840^1^.

Class: Eutardigrada Richters, 1926^65^.

Order: Parachela Schuster, Nelson, Grigarick & Christenberry, 1980^30^.

Superfamily: Isohypsibioidea Sands, McInnes, Marley, Goodall-Copestake, Convey & Linse, 2008^33^.

Family: Hexapodibiidae Cesari, Vecchi, Palmer, Bertolani, Pilato, Rebecchi & Guidetti, 2016^29^.

**Fig. 1:**
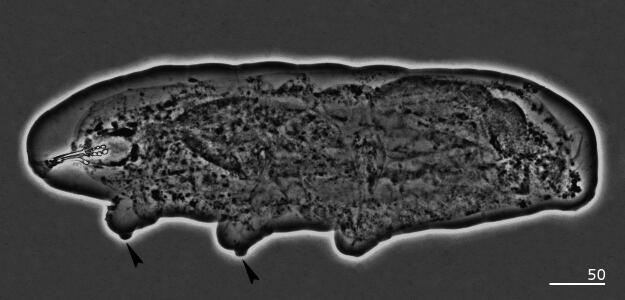
*Sinunguibius nuntius* (Ugandan population). Entire body. The black-filled indented arrowheads point to the gibbosities on legs I and II. Under PCM. Scale bars in micrometers [µm].

**Fig. 2:**
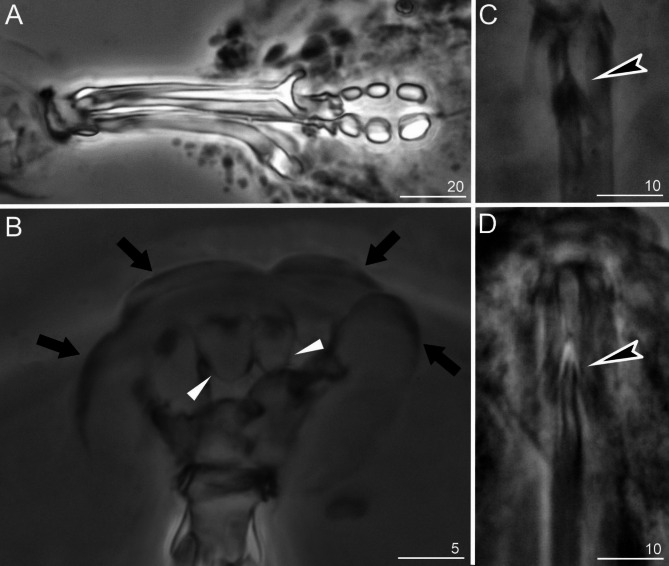
*Sinunguibius nuntius* (Ugandan population): (**A**) bucco-pharyngeal apparatus, dorso-ventral projection; (**B**) peribuccal lobes around the mouth opening indicated by black arrows and papular-shaped structures indicated by white unindented arrowheads; *Sinunguibius nuntius* (paratype): (**C**,**D**) ventral lamina indicated by black-filled indented arrowheads. All PCM. Scale bars in micrometers [µm].

**Fig. 3:**
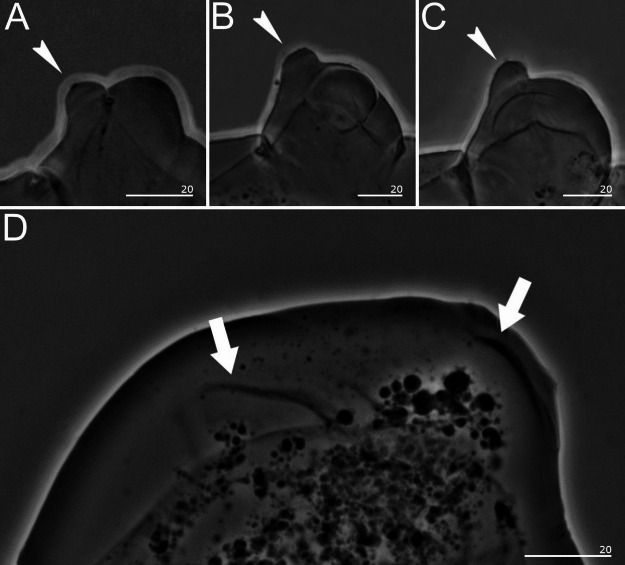
*Sinunguibius nuntius* comb. nov. (Ugandan population). (**A**) Leg I; (**B**) Leg II; (**C**) Leg III; (**D**) Legs IV. White indented arrowheads show gibbosities on legs I–III. White filled arrows indicate reduced legs IV. All PCM. Scale bars in micrometers [µm].

**Fig. 4:**
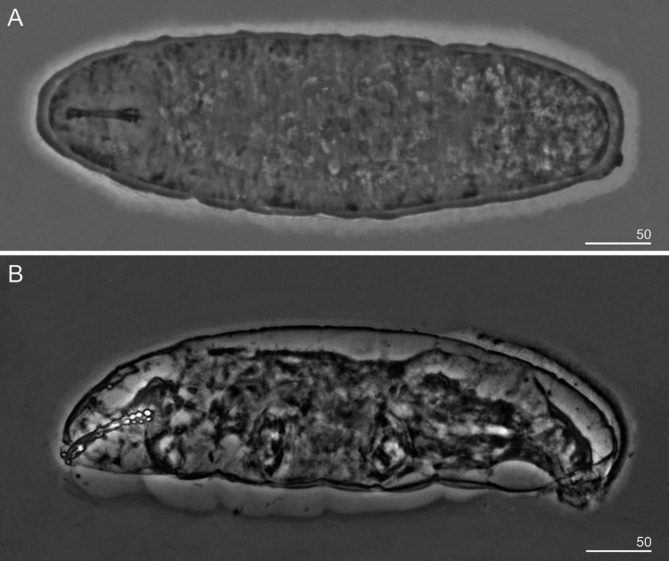
(**A**) Entire body of *Sinunguibius nuntius* comb. nov. (Paratype); (**B**) Entire body of *Sinunguibius nuntius* comb. nov. (Australian population). All PCM. Scale bars in micrometers [µm].

Genus: *Sinunguibius* gen. nov.

(Tables 1 and 2; Figs. 1, 2, 3 and 4)

**Table 1:** Measurements [in µm] and *pt* values of selected morphological structures of *Sinunguibius nuntius* comb. nov. (Ugandan population).

Character	*N*	Measurements					
		1		2		3	
		µm	*pt*	µm	*pt*	µm	*pt*
Body length		512	*–*	536	*–*	*–*	*–*
Buccal tube							
Buccal tube length		39.6	*–*	38.1	*–*	38.9	*–*
Stylet support insertion point		27.3	*68.9*	22.9	*60.0*	24.8	*63.7*
Buccal tube external width		4.0	*10.0*	5.2	*13.7*	5.4	*13.9*
Buccal tube internal width		2.7	*6.8*	3.7	*9.7*	3.9	*10.0*
Placoid lengths							
Macroplacoid 1		3.0	*7.5*	4.0	*10.4*	3.1	*8.1*
Macroplacoid 2		3.2	*8.0*	4.0	*10.4*	4.4	*11.2*
Macroplacoid 3		4.3	*10.7*	4.5	*11.8*	3.9	*10.1*
Macroplacoid row		12.2	*30.8*	14.0	*36.7*	12.3	*31.5*

“N” – number of specimens; *pt* – ratio of the length of a given structure to the length of the buccal tube expressed as a percentage.

**Table 2:** Measurements [in µm] and *pt* values of selected morphological structures of *Sinunguibius nuntius* comb. nov. From Mozambique (*Sin. Nuntius*) and Australia (*Sin. serventyi*).

Character	*Sin. nuntius* comb. nov.			*Sin. serventyi *comb. nov. syn.										
	*N*	Measuerments		*N*	Range						Mean		SD	
		µm	*pt*		µm			*pt*			µm	*pt*	µm	*pt*
Body length	1	328	*–*	6	248	–	354		*–*		294	*–*	35	*–*
Buccal tube														
Buccal tube length	1			6	30.1	–	37.0		*–*		33.9	*–*	2.8	*–*
Stylet support insertion point	1	28.8	*78.7*	5	22.5	–	28.6	*70.3*	*–*	*78.5*	26.0	*75.5*	2.2	*3.3*
Buccal tube external width	1	22.7	*14.9*	5	3.2	–	3.9	*9.6*	*–*	*11.3*	3.5	*10.6*	0.3	*0.7*
Buccal tube internal width	1	4.3	*10.5*	5	1.6	–	2.6	*5.4*	*–*	*7.6*	2.1	*6.2*	0.4	*0.9*
Placoid lengths		3.0												
Macroplacoid 1	1	2.8	*9.8*	6	2.0	–	3.4	*5.5*	*–*	*10.1*	2.8	*8.2*	0.6	*1.8*
Macroplacoid 2	1	2.4	*8.2*	6	3.0	–	3.7	*8.1*	*–*	*10.3*	3.2	*9.5*	0.3	*0.9*
Macroplacoid 3	1	2.9	*10.1*	6	3.5	–	4.8	*10.3*	*–*	*13.1*	4.0	*11.9*	0.5	*1.1*
Macroplacoid row	1	9.2	*32.0*	6	9.2	–	13.1	*29.5*	*–*	*36.8*	11.4	*33.5*	1.3	*2.8*

“N”—number of specimens/structures measured; “RANGE”—measurements taken for the smallest and the largest structure among all measured specimens; SD—standard deviation; *pt*—ratio of the length of a given structure to the length of the buccal tube expressed as a percentage.

*Genus abbreviation: Sin.*

*Type species: Sinunguibius nuntius* (Binda, 1984)^36^ comb. nov. by original designation and monotypy (Articles 68.2 and 68.3 of the ICZN, 1999).

*Etymology*: The name *Sinunguibius* is a combination of two Latin words: *sinus*, meaning “without” or “lack of” and *ungui*, meaning “claw”.

*Diagnosis*: Cuticle smooth (Figs. 1 and 4). Peribuccal papular-shaped structures (the exact number unknown) and lobes present around the mouth opening (Fig. 2B). Buccal apparatus of the *Hexapodibius* type (Fig. 2A, C, D). Ventral lamina present (Fig. 2C,D). Oral cavity armature absent or not visible under PCM. Pharynx with three macroplacoids (Figs. 1, 2 and 4). Stylet furcae typically-shaped (Fig. 2A). Claws on all legs absent (Figs. 1, 3 and 4). The first three pairs of legs with single gibbosity on each leg (Fig. 1 and 3A–C). The fourth pair of legs strongly reduced (Fig. 3D). Eggs unknown.

Genus composition: *Sinunguibius nuntius* (Binda, 1984)^36^ comb. nov.

*Differential diagnosis*: In terms of a buccal tube provided with a ventral lamina and the reduction of claws, *Sinunguibius* gen. nov. is similar to the following genera: *Necopinatum*,* Apodibius*,* Haplomacrobiotus* May, 1948^66^, *Haplohexapodibius* Pilato & Beasley, 1987^67^, *Hexapodibius*, and *Parhexapodibius* Pilato, 1969^22^. However, the new genus differs from them specifically in the following characteristics:

- *Necopinatum* by: the presence of three macroplacoids in the pharynx (two macroplacoids present in *Necopinatum*), the absence of claws on all legs (strongly reduced claws present on the legs I–III in *Necopinatum*) and the presence of a single gibbosity at the end of each leg (presence of two gibbosities on the legs I–III in the genus *Necopinatium*).
- *Apodibius* by: the presence of three macroplacoids in the pharynx (two macroplacoids present in *Apodibius*).
- *Haplomacrobiotus* by: the absence of claws on all legs (only the secondary branches are reduced in all claws in *Haplomacrobiotus*).
- *Haplohexapodibius* by: the absence of claws on all legs (only claw secondary branches reduced on first three pairs of legs, and claws absent only on hind legs in *Haplohexapodibius*).
- *Hexapodibius* by: the presence of three placoids (two or three placoids present in *Hexapodibius*), the absence of claws on all legs (strongly reduced claws but present on the first three pairs of legs in *Hexapodibius*).
- *Parhexapodibius* by: the absence of claws on all legs (claws present on all legs, strongly reduced only on the hind legs, in *Parhexapodibius*).

Species: *Sinunguibius nuntius* (Binda, 1984)^36^.

**Fig. 5:**
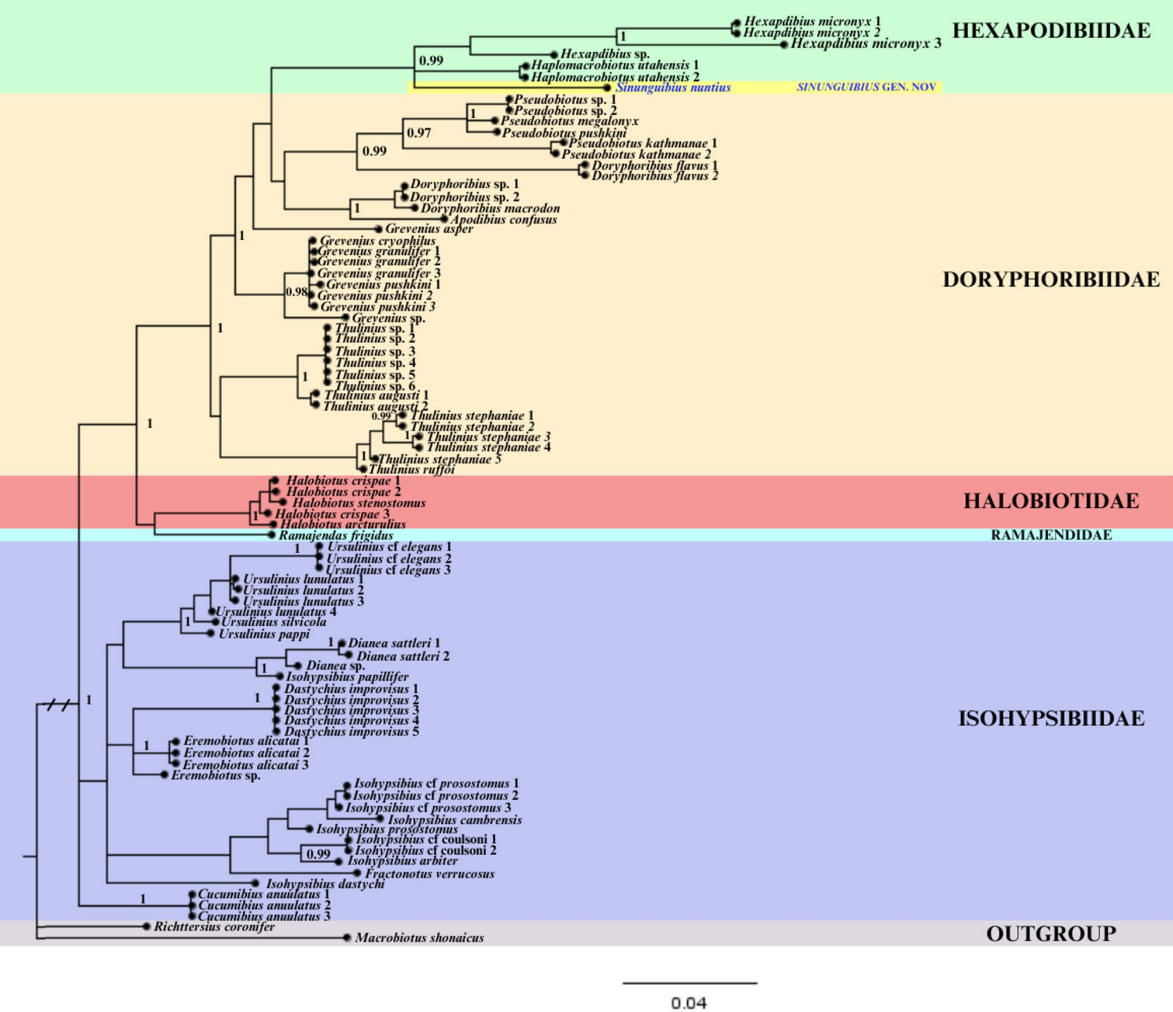
Bayesian phylogeny constructed from concatenated sequences (18S rRNA + 28S rRNA) of the superfamily Isohypsibioidea. Numbers above branches indicate Bayesian posterior probabilities (pp). pp < 0.95 are not shown. The new genus *Sinunguibius* gen. nov. is indicated in blue font. The families are indicated by colours respectively: Hexapodibiidae – light green; Doryphoribiidae – orange; Halobiotidae – red; Ramajendidae – light blue; Isohypsibiidae – purple.

Synonym: *Sinunguibius serventyi* (Morgan & Nicholls, 1986)^37^.

(Tables 1 and 2; Figs. 1, 2, 3, 4 and 5)

*Material examined*: One paratype of *Sin. nuntius*: Slide No. 3637. Six specimens of *Sin. nuntius* (Australian population): Slide No: WAM 86/34-9-53. Two specimens and one exoskeleton (after DNA extraction) from the Ugandan population mounted on microscope slides in Hoyer’s medium.

*Locality* (Ugandan population): 00°40’32.0"N 30°13’51.7"E; 1500 m asl.: Kalyango hill, Uganda; moss with soil on rock, coll. Jędrzej Warguła, 11 July 2023.

*Depositories*: The holotype and paratypes of *Sin. nuntius* are preserved in Department of Animal Biology, University of Catania (Via Androne, 81, 95124 Catania CT, Italy). Specimens from the Australian population are deposited in Western Australian Museum (49 Kew Street, Welshpool, WA 6106, Locked Bag 49, Welshpool DC, Australia). Two slides with two specimens from the Ugandan population (Slide: UG71/1 and UG71/2) are deposited at the Department of Animal Taxonomy and Ecology, Adam Mickiewicz University in Poznań (Uniwersytetu Poznańskiego 6, Poznań, Poland). One slide UG71/3 with the exoskeleton is deposited in Institute of Systematics and Evolution of Animals, Polish Academy of Sciences (Sławkowska 17, 31 − 016, Kraków, Poland).

*Species redescription*: Cuticle smooth (Figs. 1 and 4). Eyes absent in specimens fixed in Hoyer’s medium. Peribuccal papular-shaped structures (the exact number unknown) and six lobes present around the mouth opening (Fig. 2B). Buccal apparatus of the *Hexapodibius* type (Figs. 2A, C–D). The ventral lamina composed of a very narrow anterior portion, which then transitions into a larger triangular portion with two processes pointing backward and sideways (Figs. 2C–D). Oral cavity armature absent or not visible under PCM. Pharynx in elongated-oval shape with three roundish macroplacoids with configuration 1 < 2 < 3 (Figs. 1, 2 and 4). Stylet furcae typically-shaped (Fig. 2A). Claws on all legs absent (Figs. 1, 3 and 4). The first three pairs of legs with a single gibbosity on each leg (Figs. 1 and 3A–C). The fourth pair of legs strongly reduced (Fig. 3D). Eggs unknown.

*DNA sequences*: The sequences obtained for the two molecular markers analyzed in this study were of good quality.

The 18S rRNA sequence (GenBank: PV708085), 651 bp long.

The 28S rRNA sequence (GenBank: PV708086), 746 bp long.

### Phylogenetic results

A total of 83 terminal taxa representing 45 species from the superfamily *Isohypsibioidea*, were included in the phylogenetic analysis. These taxa encompassed representatives from five families^31,53^: Isohypsibiidae, Doryphoribiidae, Hexapodibiidae, Halobiotidae Gąsiorek, Stec, Morek & Michalczyk, 2019^31^, and Ramajendidae Tumanov, 2022^41^. The analysis revealed that our specimen of *Sin. nuntius* comb. nov. clustered with members of the family Hexapodibiidae, specifically genera *Haplomacrobiotus*, and *Hexapodibius*. Our results also showed a clear distinction between the clawless *Sin. nuntius* comb. nov. and also clawless eutardigrade genus *Apodibius* possessing two macroplacoids in the pharynx, which are assigned to the family Doryphoribiidae. Furthermore, our analysis indicated a grouping of Hexapodibiidae with Doryphoribiidae, rendering the latter paraphyletic. However, many internal nodes were weakly supported due to limited taxonomic and phylogenetic sampling, underscoring the need for more comprehensive analyses in future studies.

### Current status of the family Hexapodibiidae

The family Hexapodibiidae was established by Cesari et al., 2016^29^ based on distinct morphological features and molecular data. Our morphological and phylogenetic analyses confirm that the newly described genus *Sinunguibius* gen. nov. also belongs to this family, prompting revision of the diagnostic characteristics of Hexapodibiidae.

Famliy: Hexapodibiidae Cesari, Vecchi, Palmer, Bertolani, Pilato, Rebecchi & Guidetti, 2016^29^.

Composition: *Hexapodibius* (type genus), *Parhexapodibius*, *Haplomacrobiotus*, *Haplohexapodibius*, *Sinunguibius* gen nov.

*Amended description*: Bucco–pharyngeal apparatus of the *Hexapodibius* type, as defined by Pilato & Binda, 2010^47^. The stylet furcae with typical shape. Pharyngeal apophyses are present. Claws are either absent, as in *Sinunguibius* gen. nov., or strongly reduced. When present, they typically form asymmetrical diploclaws with a 2121 configuration, as observed in *Hexapodibius* and *Parhexapodibius*, or consist only of the main branches, with the secondary branch reduced, at least on legs I–III, as in *Haplomacrobiotus* and *Haplohexapodibius*. Claws of the *Hexapodibius*-type are very short, lack a common basal tract, and have a base approximately as wide as the combined width of both branches, with a clear suture separating them. *Haplomacrobiotus*-type claws consist of a single branch, sometimes featuring small spurs on the fourth pair of legs. Lunules and other cuticular thickenings are absent on legs in all known species.

### Remarks on *Sinunguibius serventyi* synonymization

Morphometric comparisons conducted in our study generally support the conclusion that *Sin. serventyi*, originally described from Australia, is a synonym to *Sin. nuntius* as previously proposed by Van Rompu et al., 1994^38^. Dimensions of most of the morphometric traits overlap between all analysed populations, with the exception of the buccal tube width (both external and internal), which is smaller in the Australian population compared to the two populations from Africa. Taking into account the considerable geographical distance between these localities (Australia vs. Africa), we emphasize that molecular data and morphological analysis under scanning electron microscope are necessary to definitively resolve their taxonomic status.

## Discussion

### Taxonomic position of *Sinunguibiusnuntius* from Uganda

Based on morphological characteristics such as the absence of claws, the presence of gibbosities, three placoids, and peribuccal structures, we classified these specimens as belonging to the species *Sinunguibius nuntius* comb. nov. The observed differences in measurements are considered insufficient to justify designation of the separate species. Considering that previous descriptions of *Sin. nuntius* were based on only a few individuals, it is not possible to reliably compare morphometric data, as the observed differences may reflect individual variation rather than taxonomic divergence.

The absence of clear morphological distinctions and the lack of genetic data support our conclusion that at present these specimens should be assigned to the known species *Sinunguibius nuntius* comb. nov.

### Remarks on the classification of *Apodibiusnuntius* from Venezuela

The population of *Apodibius nuntius* described in 2017 from Venezuela^68^ is considered a distinct case compared to the occurrence of this species in Australia. Based on morphological data, such as the markedly different shape of the macroplacoids and the presence of cuticular bars, it is unlikely that the South American population truly represents the species *Sin. nuntius*. Due to this discrepancy and the absence of genetic data, we cannot confidently assert that this taxon should be included within the newly established genus *Sinunguibius*. In this case, the final taxonomic position can only be determined based on comprehensive morphological analysis which will by supported by genetic data.

### Taxonomic position of *Sinunguibius* gen. nov 

The newly described genus *Sinunguibius* gen. nov. exhibits several traits characteristic of the family, including a *Hexapodibius*-type buccal tube and distinct peribuccal structures. These features, together with the provided phylogenetic analysis, strongly support its placement within Hexapodibiidae and reinforce the recognition of the family as a distinct and coherent taxonomic group. Our updated assessment of Hexapodibiidae reveals a progressive pattern of claw reduction, occurring to varying degrees across all its known representatives. While partial claw reduction – typically restricted to the last pair of legs – has been previously reported in genera such as *Hexapodibius* and *Haplohexapodibius*, the newly established genus *Sinunguibius* gen. nov. exhibits a complete absence of claws on all legs. This likely represents the most advanced stage of claw reduction within the family. Interestingly, such a progressive pattern of claw reduction was also noted in the genus *Xerobiotus* and commented recently by Vecchi et al., 2022^69^. These findings suggest that complete loss of claws in tardigrades is a convergent trait that has evolved independently in at least two families^24,35^. This assumption is supported by both molecular evidence and clear morphological distinctions among known clawless genera. However, the evolutionary pathways and genetic basis of this reduction remain insufficiently understood and require further study. An additional character that may prove crucial for future classification within the family Hexapodibiidae is the presence of a dual system of peribuccal structures, consisting of rings of peribuccal lobes and papular-shaped elements surrounding the mouth opening. Although this system is mentioned in taxonomic diagnoses for almost^22^ all currently described representatives of the family, the number or presence – particularly of the papular like structures – have often been overlooked. This oversight likely stems from their position and limited visibility under phase-contrast microscopy. To clarify their taxonomic significance and assess whether this character is truly consistent across the family, comprehensive morphological investigations using scanning electron microscopy are essential.

However, we remain cautious on the definitive placement of Hexapodibiidae within the superfamily Isohypsibioidea, as this issue remains unresolved and has been debated by several authors^29,30,70^. Our concerns stem primarily from the limited availability of molecular data for genera within this family, particularly the lack of such data for *Haplohexapodibius*. Notably, the ongoing debate on the family’s placement arises from inconsistencies between morphological analyses of claw structures and molecular phylogenies, which have yielded conflicting results. The controversy centres on hypothesis regarding claw evolution within Hexapodibiidae. According to Pilato, 2024^70^, the current placement of the family within Isohypsibioidea would necessitate an additional reduction step in claw evolution – specifically, the derivation of distinctly divided branches of Hexapodibiidae-type claws from the undivided claws typical of Isohypsibioidea. Such a process appears overly complex and, as argued by the author, may indicate an incorrect taxonomic assignment. Nevertheless, current molecular data suggest that the Hexapodibiidae family should remain within the Isohypsibioidea superfamily^30 ^although it should be emphasized that significantly more morphological and molecular data are needed to gain accurate insight into this issue.

Due to the absence of claws in the genus we describe and assign to Hexapodibiidae, we are unable to contribute new data to this aspect of the discussion. We suggest that resolving the phylogenetic position of Hexapodibiidae, and clarifying relationships within Isohypsibioidea as a whole, will require a more comprehensive dataset that integrates both molecular and morphological evidences.

## Conclusions

Our study documented another case of convergent evolution in claw reduction among tardigrades inhabiting soil environments. Importantly, this represents the first evidence of convergent evolution in clawless tardigrades. Interestingly, in the case of claws, elongation has also been observed. This process which, similarly to reduction, occurs in various tardigrade genera^71–83^such as *Mesobiotus* Vecchi et al., 2016^39^, *Macrobiotus* Schultze, 1834^84^, *Tenuibiotus* Pilato & Lisi, 2011^85^, *Diaforobiotus* Guidetti, Rebecchi, Bertolani, Jönsson, Kristensen & Cesari, 2016^86^, *Vladimirobius* Kaczmarek, Bartylak & Roszkowska, 2020^80^, *Weglarskobius* Kaczmarek, Bartylak & Roszkowska, 2020^80^, *Cryoconicus* Zawierucha, Stec, Lachowska-Cierlik, Takeuchi, Z. Li & Michalczyk, 2018^87^, *Ramajendas* Pilato & Binda, 1990^88^. This may indicate that, although claw morphology is considered a conservative trait with strong phylogenetic significance, the dimensions of claws appear to be a more plastic character, potentially shaped by environmental factors.

## Supplementary Information

Below is the link to the electronic supplementary material.

Supplementary Material 1

Supplementary Material 2

Supplementary Material 3

Supplementary Material 4

Supplementary Material 5

## Data Availability

The sequence data supporting the findings of this study have been deposited in GenBank under the primary accession numbers: PV708085 and PV708086.
