# Supplementary material for: Integrative taxonomy elucidates phylogenetic position of a clawless African eutardigrade (Tardigrada) supporting the erection of a new genus
Source: Sci Rep. 2025 Oct 3;15:34511. doi: 10.1038/s41598-025-17679-7 (PMC12494984; doi:10.1038/s41598-025-17679-7)
Supplement: Supplementary file 2 — Supplementary Material 2 [file 41598_2025_17679_MOESM2_ESM.docx]

**Supplementary material Table S1.**

Complete list of species analyzed, with their GenBank accession numbers and source publications. Newly sequenced species highlighted in bold.

| Species | Accession numer for 18S rRNA | Accession numer for 28S rRNA | Source |
| --- | --- | --- | --- |
| ***Sinunguibius nuntius*** | **PV708085** | **PV708086** | **This study** |
| *Apodibius confusus* | KC582830 | KC582834 | Dabert et al., 2014 |
| *Cucumibius annulatus* 1–3 | PQ069998–70000 | PQ070026–28 | Tumanov et al. 2024 |
| *Dastychius improvisus* 1–5 | MK737028–31 | MK737035–39 | Mioduchowska et al., 2021 |
| *Dianea papillifera* | EU266925 | - | Sands et al., 2008 |
| *Dianea sattleri* 1–2 | MK675926–27 | MK675915–16 | Gąsiorek et al., 2019a |
| *Dianea* sp. | PQ069995 | - | Tumanov et al. 2024 |
| *Doryphoribius flavus* 1–2 | HQ604940–41 | - | Bertolani et al., 2014 |
| *Doryphoribius macrodon* | HQ604942 | - | Bertolani et al., 2014 |
| *Doryphoribius* sp. 1–2 | PQ069996–97 | PQ070024–25 | Tumanov et al. 2024 |
| *Eremobiotus alicatai* 1–3 | HQ604951–53 | - | Bertolani et al., 2014 |
| *Eremobiotus sp* | MK675928 | MK675917 | Gąsiorek et al., 2019a |
| *Fractonotus verrucosus* | MG800855 | MG800856 | Gąsiorek et al., 2019b |
| *Grevenius asper* | MZ050453 | MZ050456 | Tumanov et al. 2024 |
| *Grevenius cryophilus* | MW012741 | MW012740 | Zawierucha et al., 2020 |
| *Grevenius granulifer* 1 | KT778603 | - | Cesari et al., 2016 |
| *Grevenius granulifer* 2 | EF620403 | - | Møbjerg et al., 2007 |
| *Grevenius granulifer* 3 | PQ275724 | PQ275726 | Gąsiorek et al., 2024 |
| *Grevenius pushkini* 1 | MK675929 | MK675918 | Gąsiorek et al., 2019a |
| *Grevenius pushkini* 2–3 | PQ070001–02 | PQ070030 | Tumanov et al. 2024 |
| *Grevenius* sp. | PQ070003 | - | Tumanov et al. 2024 |
| *Halobiotus arcturulius* | MZ050455 | MZ050452 | Tumanov, 2022 |
| *Halobiotus crispae* 1–2 | EF620401–02 | - | Møbjerg et al., 2007 |
| *Halobiotus crispae* 3 | PQ070004 | PQ070031 | Tumanov et al. 2024 |
| *Halobiotus stenostomus* | AY582121 | - | Jorgensen & Kristensen, 2004 |
| *Haplomacrobiotus utahensis* 1–2 | KT778600–01 | - | Cesari et al., 2016 |
| *Hexapodibius micronyx* 1–2 | HQ604915–16 | - | Bertolani et al., 2014 |
| *Hexapodibius micronyx* 3 | MK675930 | - | Gąsiorek et al., 2019a |
| *Hexapodibius* sp*.* | HQ604917 | - | Bertolani et al., 2014 |
| *Isohypsibius arbiter* | KT778602 | - | Cesari et al., 2016 |
| *Isohypsibius cambrensis* | AM500652 | - | Kiehl et al., 2007 |
| *Isohypsibius* cf. *coulsoni* 1–2 | PQ070005–06 | PQ070032–33 | Tumanov et al. 2024 |
| *Isohypsibius dastychi* | HQ604954 | - | Bertolani et al., 2014 |
| *Isohypsibius prosostomus* | EF620404 | - | Møbjerg et al., 2007 |
| *Isohypsibius* cf. *prosostomus* 1–3 | PQ070007–09 | PQ070034–36 | Tumanov et al. 2024 |
| *Pseudobiotus kathmanae* 1–2 | HQ604957–58 | - | Bertolani et al., 2014 |
| *Pseudobiotus megalonyx* | MK675931 | MK675920 | Gąsiorek et al., 2019a |
| *Pseudobiotus* sp. 1–2 | PQ070010–11 | PQ070037–38 | Tumanov et a., 2024 |
| *Ramajendas frigidus* | MZ050453 | MZ050450 | Tumanov, 2022 |
| *Thulinius augusti* 1–2 | KF360230–31 | - | Bertolani et al., 2014 |
| *Thulinius ruffoi* | MK675932 | MK675921 | Gąsiorek et al., 2019a |
| *Thulinius* sp. 1–6 | PQ070012–17 | PQ070039–44 | Tumanov et al. 2024 |
| *Thulinius stephaniae* 1–4 | GQ925698–701 | - | unpublished |
| *Thulinius stephaniae* 5 | AF056023 | - | Garey et al., 1999 |
| *Ursulinius* cf. *elegans* 1–3 | PQ070021–23 | PQ070048–50 | Tumanov et al. 2024 |
| *Ursulinius lunulatus* 1–3 | PQ070018–20 | PQ070045–47 | Tumanov et al. 2024 |
| *Ursulinius lunulatus* 4 | MK675933 | MK675922 | Gąsiorek et al., 2019 |
| *Ursulinius pappi* | MK675934 | MK675923 | Gąsiorek et al., 2019 |
| *Ursulinius silvicola* | MK675935 | - | Gąsiorek et al., 2019 |
| *Macrobiotus shonaicus* | MG757132 | MG757133 | Stec et al., 2018 |
| *Richtersius coronifer* | MH681760 | MH681757 | Stec et al., 2020 |

References

Bertolani, R., Guidetti, R., Marchioro, T., Altiero, T., Rebecchi, L., Cesari, M. Phylogeny of Eutardigrada: New molecular data and their morphological support lead to the identification of new evolutionary lineages, *Mol. Phyl. Evol.* **76,** 110–126; 10.1016/j.ympev.2014.03.006 (2014).

Cesari, M. et al. What if the claws are reduced? Morphological and molecular phylogenetic relationships of the genus *Haplomacrobiotus* May, 1948 (Eutardigrada, Parachela). *Zool. J. Linn. Soc.* **178**, 819–827; 10.111/zoj.12424 (2016).

Dabert, M., Dastych, H., Hohberg, K., & Dabert, J. Phylogenetic position of the enigmatic clawless eutardigrade genus *Apodibius* Dastych, 1983 (Tardigrada), based on 18S and 28S rRNA sequence data from its type species *A. confusus*. *Mol. Phyl. Evol.* **70**, 70–75; 10.1016/j.ympev.2013.09.012 (2014).

Gąsiorek, P. *Grevenius granulifer* (Thulin, 1928) revised: a fresh look at one of the most intensively studied water bears (Eutardigrada: Isohypsibioidea). *Org. Divers. Evol.* **25**, 29–41; 10.1007/s13127-024-00658-7 (2024).

Gąsiorek, P., Stec, D., Morek, W. & Michalczyk, Ł. Deceptive conservatism of claws: distinct phyletic lineages concealed within Isohypsibioidea (Eutardigrada) revealed by molecular and morphological evidence. *Contrib. Zool.* **88**, 78–132; 10.1163/18759866-20191350 (2019a).

Gąsiorek, P., Morek, W., Stec, D., Blagden, B., & Michalczyk Ł. Revisiting Calohypsibiidae and Microhypsibiidae: *Fractonotus* Pilato, 1998 and its phylogenetic position within Isohypsibiidae (Eutardigrada: Parachela). *Zoosystema*, **41,** 71–89; 10.5252/zoosystema2019v41a6 (2019b).

Garey, J. R., Nelson, D. R, Mackey, L. Y., & Li, J. Tardigrade Phylogeny: Congruency of Morphological and Molecular Evidence. *Zool. Anz.* **238**, 205–210 (1999).

Jørgensen, A. & Kristensen, R. M. Molecular phylogeny of Tardigrada – investigation of the monophyly of Heterotardigrada. *Mol. Phyl. Evol.* **32**, 666–670. doi:10.1016/j.ympev.2004.04.017 (2004).

Kiehl, E., Dastych, H., D’Haese, J. & Greven, H. The 18S rDNA sequences support polyphyly of the Hypsibiidae (Eutardigrada). *J. limn*. **66**, 21–25; 10.4081/jlimnol.2007.s1.21 (2007).

Mioduchowska, M., Kačarević, U., Miamin, V., Giginiak, Y., Parnikoza, I., Roszkowska, M., & Kaczmarek, Ł. Redescription of Antarctic eutardigrade *Dastychius improvisus* (Dastych, 1984) and some remarks on phylogenetic relationships within Isohypsibioidea. *E. Zool. J.* **88**, 117–131; 10.1080/24750263.2020.1854877 (2021).

Møbjerg, N., Jørgensen, A., Eibye-Jacobsen, J., Halberg, K. A., Persson, D. & Kristensen, R. M. New records on cyclomorphosis in the marine eutardigrade *Halobiotus crispae* (Eutardigrada: Hypsibiidae). *J. Limnol.* **66,** 132–140 (2007).

Sands, C. J. et al. Phylum Tardigrada: an "individual" approach. *Cladistics* **24(6)**, 861–871; 10.1111/j.1096-0031.2008.00219.x (2008).

Stec, D., Arakawa, K., & Michalczyk, Ł.. An integrative description of *Macrobiotus shonaicus* sp. nov. (Tardigrada: Macrobiotidae) from Japan with notes on its phylogenetic position within the hufelandi group. *PLoS ONE*, **13(2)**, 10.1371/journal.pone.0192210 (2018).

Stec, D., Krzywański, Ł., Arakawa, K., & Michalczyk, Ł. A new redescription of *Richtersius coronifer*, supported by transcriptome, provides resources for describing concealed species diversity within the monotypic genus *Richtersius* (Eutardigrada). *Zool. Lett.* **6**, 2; 10.1186/s40851-020-0154-y (2020).

Tumanov, D. V. End of a mystery: Integrative approach reveals the phylogenetic position of an enigmatic Antarctic tardigrade genus *Ramajendas* (Tardigrada, Eutardigrada). *Zool. Scr.*, **51**, 217–231; 10.1111/zsc.12521 (2022).

Tumanov, D., Shunatova, N., Fedyuk, K. Integrative description of Grevenius annulatus (Eutardigrada, Isohypsibioidea) from North-West Russia with new data on the species cuticular structure leads to the institution of a new genus. *Zool. Scr.* 10.1111/zsc.12703 (2024).

Zawierucha, K., Buda, J., Novotna Jaromerska, T., Janko, K., & Gąsiorek, P. Integrative approach reveals new species of water bears (*Pilatobius*, *Grevenius*, and *Acutuncus*) from Arctic cryoconite holes, with the discovery of hidden lineages of *Hypsibius*. *Zool. Anz.* **289**, 141–165; 10.1016/j.jcz.2020.09.004 (2020).
